# Regulation of regulatory T cells in cancer

**DOI:** 10.1111/imm.13064

**Published:** 2019-05-22

**Authors:** Julie Stockis, Rahul Roychoudhuri, Timotheus Y. F. Halim

**Affiliations:** ^1^ CRUK Cambridge Institute University of Cambridge Cambridge UK; ^2^ Laboratory of Lymphocyte Signalling and Development The Babraham Institute Cambridge UK

**Keywords:** cancer, chemokine/chemokine receptors, cytokines/cytokine receptors, regulatory T cells, tumour immunology

## Abstract

The inflammatory response to transformed cells forms the cornerstone of natural or therapeutically induced protective immunity to cancer. Regulatory T (Treg) cells are known for their critical role in suppressing inflammation, and therefore can antagonize effective anti‐cancer immune responses. As such, Treg cells can play detrimental roles in tumour progression and in the response to both conventional and immune‐based cancer therapies. Recent advances in our understanding of Treg cells reveal complex niche‐specific regulatory programmes and functions, which are likely to extrapolate to cancer. The regulation of Treg cells is reliant on upstream cues from haematopoietic and non‐immune cells, which dictates their genetic, epigenetic and downstream functional programmes. In this review we will discuss how Treg cells are themselves regulated in normal and transformed tissues, and the implications of this cross talk on tumour growth.

## Introduction

The interplay between cancer and inflammation is noteworthy in both its importance and its complexity. It is now appreciated that the immune system has a critical importance in both carcinogenesis and tumour rejection. Whereas inflammation can contribute to carcinogenesis and thereby serve a deleterious role, the effect of immune activation during tumour progression is often beneficial and can promote tumour rejection. Although superficially paradoxical, this disparity can be explained by differences in disease stage, location and host, among many other factors.^1^ Nevertheless, it is generally accepted that certain inflammatory signatures correspond with either pro‐ or anti‐tumorigenic potential. Cytotoxic lymphocytes, such as natural killer or CD8^+^ T cells, or type 1 helper CD4^+^ T (Th1) cells, have potent direct and indirect anti‐cancer tumour activity and their presence in human tumours is frequently associated with favourable outcomes (reviewed in ref. 2). In contrast, regulatory T (Treg) cells suppress the function of conventional T (Tconv) cells including CD4^+^ and CD8^+^ T cells and although this function is required to prevent unwanted autoimmune and allergic inflammation, it is now known that Treg cells play a critical role in suppressing immune responses in cancer.^3^, ^4^ Treg cells also have a number of non‐classical functions, some of which may directly influence tumour cell biology, or act in a tissue‐specific manner. Hence, it is imperative to resolve the role of Treg cells at each distinct stage of cancer, from carcinogenesis to tumour progression and metastasis. In this review we will focus on the known and proposed mechanisms that regulate the recruitment, local homeostasis, and function of Treg cells during cancer progression.

### Identification of Treg cells

Treg cells are a suppressive subset of CD4^+^ T cells required to prevent lethal inflammation.^5^, ^6^ In mice, Treg cells arising early in neonatal life are required for tolerance to self‐antigens, a conclusion made from the paradoxical observation that neonatal thymectomy results in a lethal inflammatory disorder, that is reversible by the transfer of CD4^+^ CD25^+^ Treg cells.^7^ Treg cells are dependent upon the lineage‐specifying transcription factor Foxp3 and mice and humans carrying inactivating mutations of Foxp3 succumb to lethal inflammatory disease within approximately 3 weeks and in early infancy, respectively.^8^, ^9^, ^10^, ^11^, ^12^, ^13^, ^14^ Whereas Foxp3 expression is predominantly restricted to Treg cells in mouse, FOXP3 is expressed in both Treg and Tconv cells upon acute activation in humans.^15^, ^16^, ^17^ Hence, although Foxp3 expression usefully distinguishes murine Treg cells from CD4^+^ Tconv cells, human FOXP3^+^ T cells may comprise both Treg and Tconv cells, especially during ongoing immune responses. Instead, demethylation of the intronic *Foxp3 cis*‐regulatory element *CNS2* (conserved non‐coding sequence 2) is a cardinal feature of Treg cells, both in human and mouse.^18^, ^19^

### Dual ontogeny of Treg cells

Thymus‐derived (tTreg) cells develop in response to high‐affinity interactions between the T‐cell receptor (TCR) of double‐positive and CD4 single‐positive thymocytes and self‐peptide–major histocompatibility complex (MHC) complexes in the thymus, and, among other functions, suppress autoimmune reactions directed against self‐antigens. As a product of this selection process in the thymus, tTreg cells have a largely distinct TCR repertoire to conventional CD4^+^ T cells.^20^, ^21^ Thymic selection results in differentiation of Treg cells with specificity for self‐antigens, but tolerance to innocuous foreign antigens unanticipated in the thymus is mediated by peripheral Treg (pTreg) cells, induced in peripheral tissues.^22^ This occurs when antigens are encountered by naive CD4^+^ T cells in the absence of optimal co‐stimulation or in the presence of transforming growth factor‐*β* (TGF‐*β*) abundant at mucosal sites but also within tumours. As a result, pTreg cells prevent inflammation directed against innocuous antigens often found at mucosal sites, including antigens expressed by commensal microflora or dietary components. Whereas tTreg and pTreg cell development have important similarities, such as their dependency upon the activity of the transcription factors FOXP3 and BACH2,^23^, ^24^, ^25^, ^26^ their distinct ontogeny is reflected in differences in gene regulatory mechanisms underlying their development. This is best illustrated by the finding that an intronic *Foxp3 cis*‐regulatory element, *CNS1*, which contains SMAD3 binding sites, is necessary for pTreg cell differentiation but dispensable for tTreg cell differentiation.^27^ Although deletion of *Foxp3* leads to loss of both tTreg and pTreg cells and to lethal multi‐organ auto‐inflammation, selective ablation of the pTreg cell pool resulting from deletion of the *CNS1* locus only leads to a mild late‐onset mucosal type 2 inflammation in the gut and the lungs.^28^ Additionally, the TCR specificity of tTreg cells and pTreg cells is largely distinct.^22^, ^29^

### Functional specialization of Treg cells

Treg cells suppress inflammation through a number of mechanisms and it is now apparent that Treg cells undergo functional specialization to share some of the molecular characteristics of the cell types that they control.^30^, ^31^ For example, expression of the Th1 cell‐associated transcription factor, T‐bet, promotes Treg‐mediated restraint of type 1 inflammation.^32^, ^33^ Similarly, Treg cells expressing other CD4^+^ helper T lineage‐specific transcription factors such as ROR‐*γ*t, GATA3 and IRF4 exert specialized functions suited to suppression of inflammation driven by their cognate Th cell counterparts.^34^, ^35^, ^36^, ^37^ It is notable that despite expressing lineage‐specifying transcription factors in some cases required for production of helper cytokines by Th cells, Treg cell subsets are configured to suppress these cell types. Mechanisms by which specialized Treg cell subsets suppress their cognate T helper counterparts are unclear, expression of lineage‐specifying transcription factors such as T‐bet or GATA3 may drive Treg cells to express a similar spectrum of chemokine receptors permitting more effective co‐localization with cognate Th cells. It may also promote a similar responsiveness to environmental cues as their cognate Th counterparts.

### Non‐classical functions of Treg cells

Recent research has uncovered a pleiotropy of non‐classical mechanisms by which Treg cells contribute to homeostasis with diverse roles in physiological processes as control of tissue metabolism, stem cell maintenance and wound healing. It is significant that these processes also play fundamental roles in cancer pathophysiology. For instance, pioneering work on adipose tissue Treg cells, which express the transcription factor PAPR *γ*, demonstrate that these resident cells play critical roles in controlling tissue metabolism and insulin sensitivity.^38^, ^39^ Importantly, adipose tissue Treg cells exert their function in concert with a number of other tissue‐resident immune cells, such as macrophages, and group 2 innate lymphoid cells (reviewed in ref. 40). Other tissues, such as the muscle, harbour similar tissue‐resident Treg cell populations, which, upon detection of tissue‐damage, activate tissue‐regenerative programmes.^41^ Skin Treg cells have also been observed in close association with the stem‐cell‐containing dermal follicular regions.^42^ Surprisingly, Treg cells were in dynamic equilibrium with hair‐regrowth phases, and influenced follicular stem cell quiescence by expression of the Notch1 ligand Jagged 1. Similarly, Treg cells play an active role in protecting the intestinal epithelial, and bone marrow stem cell niche.^43^, ^44^ Finally, tissue‐resident Treg cells also express certain genes involved in epithelial cell repair, such as the growth factor amphiregulin (*Areg*),^41^, ^45^ and thereby contribute to wound healing. Hence, our appreciation of Treg cell biology is rapidly evolving, and it is likely that ‘non‐classical’ functions of Treg cells are co‐opted by cancer.

## Regulation of Treg cells in cancer

### Origin of Treg cells within tumours

**Figure 1:**
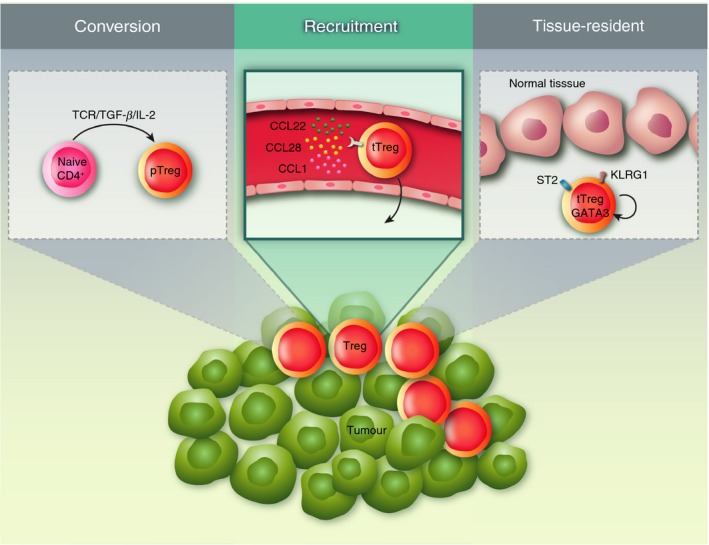
Origin of regulatory T (Treg) cells in tumours. Depicted are the three scenarios – not mutually exclusive – that could account for the presence of Treg cells within tumours. Left, conversion of naive CD4^+^ T cells into peripheral Treg (pTreg) cells; centre, recruitment of thymus‐derived Treg (tTreg) cells from the circulation; right, expansion of tissue‐resident Treg cells.

In human tumours, the frequency of FOXP3^+^ cells relative to total CD3^+^ T cells or CD8^+^ T cells is negatively correlated with survival in multiple cancer types, including renal cell carcinoma,^46^ non‐small‐cell lung carcinoma,^47^ hepatocellular carcinoma,^48^ pancreatic cancer,^49^ gastric cancer,^50^ cervical cancer,^51^ ovarian cancer,^52^, ^53^ breast cancer^54^ and colorectal cancer.^55^ The frequency of Treg cells as a ratio of total CD4^+^ T cells can be extremely high – as high as 60–80% of total CD4^+^ T cells in murine orthotopic B16 tumours where Treg cells can be unambiguously defined using intranuclear Foxp3 staining.^56^ The size of Treg cell populations within tumours can be affected by a number of processes: the conversion of conventional CD4^+^ Foxp3^−^ (Tconv cells) into pTreg cells under the influence of tumour‐derived factors including TGF‐*β*, recruitment of Treg cells from the periphery into tumours, the rate of proliferation and survival of recruited, peripherally induced or tissue‐resident Treg cells (Fig. 1). We will hereafter review the evidence in favour or against each of these hypotheses.

#### Conversion of Tconv into pTreg cells

An initial study by Valzasina *et al*.^57^ revealed that CD4^+^ CD25^−^ transferred into tumour‐bearing hosts could convert into Foxp3^+^ Treg cells within tumours. In addition, conditioned media from murine tumour cells could convert CD4^+^ CD25^−^ in CD4^+^ CD25^+^ Foxp3^+^ T cells.^58^ Because a blocking antibody to TGF‐*β* reversed this effect, the authors concluded that the secretion of TGF‐*β* by tumour cells promotes an environment favourable to the peripheral conversion of Tconv cells into tolerogenic pTreg cells. Along the same lines, the group of Zitvogel showed that rodent tumour cells induced the production of TGF‐*β* by immature myeloid dendritic cells that in turn sustain the proliferation of Treg cells.^59^ Similarly, myeloid‐derived suppressor cell‐derived interleukin‐10 (IL‐10) and TGF‐*β* supports Treg cell development both *in vitro* and in experiments where myeloid‐derived suppressor cells and T cells are adoptively transferred into irradiated tumour‐bearing mice.^60^

Recently, expression of the surface protein Neuropilin‐1 and the transcription factor Helios have been proposed to distinguish tTreg cells from pTreg cells,^61^, ^62^ although whether expression of these markers faithfully reports tTreg cells has been called into question.^63^, ^64^ This may explain contradictory reports regarding the relative frequency of pTreg and tTreg cells assessed using these markers in transplantable tumour models in mice.^65^, ^66^, ^67^ TCR repertoire analyses of mouse and human tumour‐infiltrating lymphocytes have been proposed to provide evidence of a low frequency of Tconv to pTreg conversion within tumours, as the TCR repertoire of tumour‐infiltrating Treg cells was found to be largely distinct from that of tumour‐infiltrating Tconv cells.^68^, ^69^, ^70^, ^71^, ^72^ However, the efficiency of pTreg induction is affected by antigen dose in addition to cytokine signalling^73^ and it is not inconceivable that pTreg cells arising from Tconv cells could have a substantially skewed repertoire to Tconv cells, having expanded from a small fraction of naive CD4^+^ T cells among the total Tconv pool.

Hence, it has been difficult to precisely discern the role of pTreg cells in Treg‐mediated tumour immunosuppression, although comparison of tumour growth and immune infiltrates in wild‐type and *Foxp3 CNS1* knockout mice should allow the functional contribution of pTreg cells to be defined. However, it has been proposed that decreased susceptibility of mice lacking T‐cell intrinsic expression of all three isoforms of the oxygen‐sensing prolyl hydroxylase family is attributable to defective pulmonary pTreg cell differentiation.^74^

Evidence that tTreg cells can contribute to tumour immunosuppression is much clearer. Malchow *et al*.^72^ used an autochthonous mouse model of prostate cancer driven by the restricted expression of the SV40 oncogene in the prostate to study the TCR reactivity of tumour‐associated Treg cells. On a fixed TCR‐*β* background, TCR‐*α* sequencing revealed that one TCR‐*α* sequence was recurrently expressed by Treg cells isolated from tumours of distinct animals, suggesting that Treg cells of a single specificity are recurrently enriched within these prostate tumours. They further showed that this sequence was only overrepresented in the prostate tumour and draining lymph node, but not in the spleen or the thymus, in line with the idea of a tumour‐specific enrichment. Repertoire analysis also confirmed the absence of overlap between Tconv and Treg cells isolated from these tumours. Interestingly, transgenic expression of this TCR on a *Rag1* ^−/−^ background led to the spontaneous accumulation of activated transgenic T cells in the prostate and draining lymph nodes of male tumour‐free mice, whereas in female mice transgenic T cells showed no signs of activation. Further analysis showed that thymic development of Treg cells expressing the transgenic TCR was dependent on the autoimmune regulator Aire. Altogether, these results clearly demonstrate that pre‐existing tTreg cells reactive to self‐antigens are expanded upon tumorigenesis.

#### Recruitment and retention of Treg cells to tumours

Identification of the chemokines and their cognate receptors involved in specific Treg cell recruitment and/or retention into tumour lesions is an active area of research, given their potential as druggable targets. In human ovarian cancer, Curiel *et al*.^53^ showed that, similar to blood CD4^+^ CD25^+^ Treg cells, CD4^+^ CD25^+^ T cells isolated from malignant ascites express the chemokine receptor CCR4. Furthermore, the authors found that CCL22, the ligand of CCR4, is highly expressed in the ascites and tumour tissue of patients with ovarian cancer in comparison with control groups. They demonstrated with *in vitro* migration assays that CD4^+^ CD25^+^ Treg cells migrate to malignant ascites, and that this effect is abolished upon addition of an anti‐CCL22 antibody. Other authors later reported the same observation in prostate, breast and gastric tumours.^75^, ^76^, ^77^ In the context of cancer, CCL22 is probably derived from activated myeloid cells, although lymphocytes and tumour cells are potential alternative sources.^78^, ^79^, ^80^ Upstream, CCL22 is strongly induced by IL‐4 and IL‐13, which can be produced by adaptive and innate lymphoid cells, as well as some myeloid cell types.^81^ CCL17, another ligand for CCR4, is also implicated as a chemoattractant in cancer,^76^ although it is suggested that, due to different affinities for conformational isoforms of CCR4 leading to distinct signalling characteristics, CCL17 is more important in recruiting CCR4^+^ effector T cells.^82^, ^83^ Additionally, the Sakaguchi group further ascribed high CCR4 expression in human T cells to a subset of effector Treg cells, defined as FOXP3^hi^ CD45RA^−^, in both melanoma tissues and peripheral blood.^84^ *Ex vivo* depletion of CCR4^+^ cells using an anti‐CCR4 monoclonal antibody selectively ablated effector Treg cells in healthy donors and individuals with melanoma, and was associated with an increase in CD4^+^ and CD8^+^ T‐cell responses to the cancer‐germline antigen NY‐ESO‐1 upon *in vitro* re‐stimulation. Furthermore, *in vivo* administration of Mogamulizumab, a CCR4‐depleting monoclonal antibody, in two adults with T‐cell leukaemia‐lymphoma diminished the percentage of blood effector Treg cells. In one patient whose leukaemic cells expressed NY‐ESO‐1, the reduction in effector Treg cells was further associated with an enhanced NY‐ESO‐1‐specific CD8^+^ T‐cell response.

Apart from CCL22, CCL28 produced by ovarian tumour cells under hypoxia has also been implicated in the preferential recruitment of Treg cells, both in *in vitro* migration assays using mouse and human Treg cells and in an *in vivo* model of ascitic ovarian tumours.^85^ CCR10, rather than CCR3, was shown to be the receptor involved in this effect.

In human breast cancer, RNA‐seqencing analyses of tumour‐infiltrating CD4^+^ CD25^+^ Treg cells revealed high expression of the genes encoding the chemokine receptors *CCR5*,* CCR8*,* CCR10*,* CX3CR1*,* CXCR3* and *CXCR6* compared with blood CD4^+^ CD25^+^ Treg cells.^70^ Among these, some were also shared with tumour‐infiltrating Tconv cells (*CCR5*,* CXCR3* and *CXCR6*), suggesting that Treg cells may employ both unique and shared pathways to migrate to breast tumour lesions. The authors further focused on CCR8, and showed that its surface expression was restricted to Treg cells and to a subset of natural killer T cells among CD45^+^ and non‐CD45 cells within tumours. CCR8 expression was shared by Treg cells infiltrating breast, lung and colorectal cancers, as well as in melanoma and in angiosarcoma.^70^, ^86^ Functionally, sorted tumour‐infiltrating CD4^+^ CD25^+^ Treg cells were able to migrate more robustly than Tconv cells towards the CCR8 ligand CCL1.^70^ Consistent with its tumour‐specific pattern of expression, robust *CCR8* expression by Treg cells was shown to require TCR engagement as well as soluble tumour‐derived factors using tumour explant co‐cultures. Finally, analysis of the breast samples from the The Cancer Genome Atlas data sets revealed a strong association of *CCR8* mRNA amounts normalized to *FOXP*3, but not *FOXP3* mRNA amounts alone, with poor prognosis, suggesting a detrimental role for CCR8‐expressing Treg cells in breast cancer progression.

High endothelial venules (HEV) which can be associated with tertiary lymphoid structures are present within tumours. The presence of HEV is promoted by activation of Tconv cells and dependent upon tumour necrosis factor receptor signalling.^87^ As HEV themselves drive further T‐cell recruitment, it has been proposed that this supportive relationship between Tconv cells and HEV forms the potential for a self‐amplifying loop that can drive tumour destruction. HEV neogenesis is indirectly inhibited by Treg cells through their suppression of Tconv cell activation.^87^ However, tertiary lymphoid structures contain Treg cells^88^ and whether and how HEV play an active role in recruitment of Treg cells is unclear. L‐selectin expression is required for appropriate trafficking and tissue distribution of Treg cells under physiological conditions.^89^ Whereas a majority of Treg cells in tumours are activated and express low levels of L‐selectin, it is possible that L‐selectin^+^ Treg cells are recruited by tumour HEV, which subsequently down‐regulate L‐selectin expression in response to the activating environment of the tumour. This may represent a physiological regulatory feedback mechanism in inflamed tissues that is co‐opted by tumours to counteract an otherwise beneficial self‐reinforcing feedback loop of Tconv‐driven HEV neogenesis in cancer, which could be subject to therapeutic intervention.

It is worth noting that the pathways described above may not only account for Treg cell recruitment but also for their retention within the tumour, thanks to the continuous secretion of chemo‐attractants by either tumour cells or their associated stroma. In addition, recent evidence suggests that the early activation marker CD69 could play a role in Treg cell retention within tumours, as a large proportion of tumour‐infiltrating Treg cells express high levels of this protein.^90^ Indeed, CD69 has been shown to be crucial for T‐cell trafficking by interfering with the expression of S1P1, so preventing lymphocyte egress from peripheral tissues.^91^, ^92^ Interestingly, CD69 is also linked to Treg cell function as CD69‐deficient Treg cells display an altered suppressive function *in vitro* and *in vivo*.^90^, ^93^ How CD69 affects Treg cell retention and function at the molecular level remains to be addressed.

There is now evidence to support the rationale of targeting chemokine receptors to alter Treg cell accumulation and/or retention in several types of tumours. However, it is worth noting that these pathways may also be shared with Tconv,^94^ and may not be unique to cancer, providing a potential for on‐target side effects. Moreover, the mechanisms driving chemokine receptor expression among Treg cells are unclear but there may be considerable overlap with the mechanisms that drive such receptor expression among cognate T helper counterparts, including the involvement of lineage‐specifying transcription factors of the T helper lineages that are also expressed in Treg cells.

#### Local expansion of tissue‐resident Treg cells

Recent developments in the field have led to the emerging idea that tissue‐resident Treg cells may contribute to the accumulation of Treg cells seen within tumour lesions. Although some markers of tissue‐resident Treg cells appear to be present in Treg cells in multiple tissues, large‐scale transcriptional analyses also suggest that tissue‐resident Treg cells within distinct tissues have unique phenotypes.^95^, ^96^ Pan‐tissue tissue‐resident Treg markers include the IL‐33 receptor ST2 (encoded by gene *Il1rl1*), the activation marker KLRG1, the transcription factor GATA‐3 and the growth factor Amphiregulin.

In searching for a potential role of tissue‐resident Treg cells in tumour progression, Green *et al*.^97^ analysed transplantable lung tumours in mice and found that tumour‐infiltrating Treg cells expressed higher levels of Amphiregulin compared with normal lung Treg cells. In one of the two lung tumour models tested, conditional deletion of *Areg* in Treg cells resulted in a decreased tumour volume. Although the authors excluded both immune and tumour cells, the precise identity of the cells targeted by the Treg‐derived Amphiregulin remains to be addressed. In line with these data, an increased proportion of Treg cells were shown to be ST2^+^ in both primary orthotopic mouse mammary carcinoma and lung metastases, and ST2^+^ lung tumour Treg cells, but not ST2^−^, were shown to produce Amphiregulin.^98^

In human breast tumour and healthy adjacent tissue, RNA‐sequencing analyses from the Plitas *et al*.^70^ study described above revealed that the gene expression profile of tumour‐infiltrating Treg cells was similar to those of the tissue‐resident Treg cells of the normal breast parenchyma, but distinct from the profile of the CD45RO^+^ activated Treg cells isolated from peripheral blood, used here as a reference of activated Treg cells. Surprisingly though, extraction of the TCR repertoire of these cells from RNA‐sequencing data did not support the hypothesis of local tissue‐resident Treg cell expansion: only low clonal overlap was found between the tumour‐infiltrating Treg cells and those from the normal adjacent tissue.

Altogether, there is not much evidence supporting the hypothesis of the local amplification of tissue‐resident Treg cells within tumour lesions. However, this does not imply that they do not make a significant contribution to tumour progression. Alternatively, their pre‐existence in the tissue‐of‐origin of cancer may position them to contribute to early events in carcinogenesis and metastasis through classical or non‐classical functions. Further studies will be needed to address these hypotheses.

### Signals regulating Treg cells within tumours

#### Co‐stimulatory and co‐inhibitory receptors

**Figure 2:**
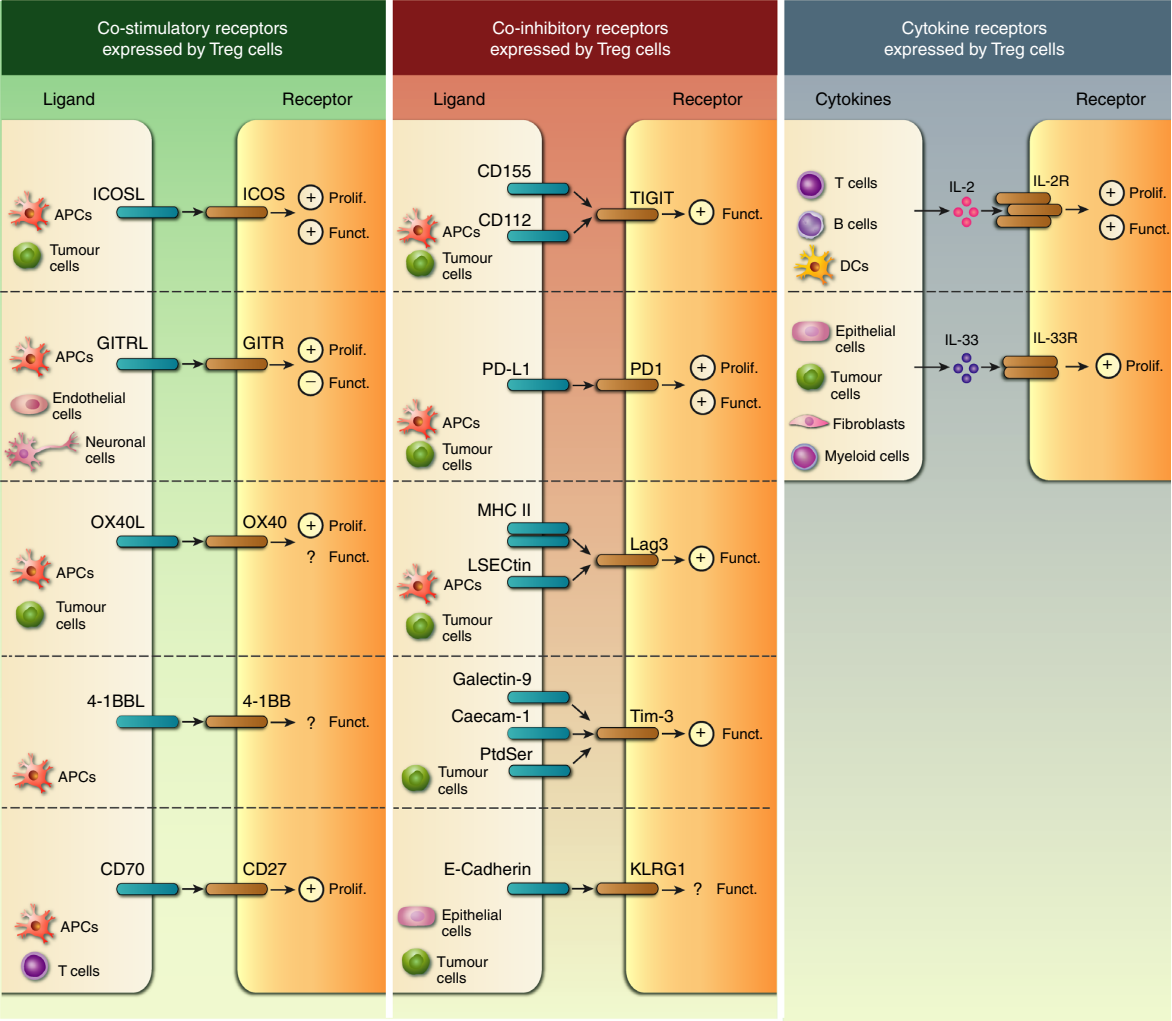
Co‐stimulatory, co‐inhibitory and cytokine receptors expressed by regulatory T (Treg) cells in tumours. Co‐stimulatory (left) and co‐inhibitory (middle) molecules, as well as cytokine receptors (right), deliver signals to Treg cells following engagement by their ligands expressed by immune and/or non‐immune cell types within the tumour. Positive and negative signs in the Treg cell indicate either a positive effect on proliferation (Prolif.) or function (Funct.) or an inhibitory effect (i.e. reduced function), respectively, after receptor engagement. Question marks (?) indicate that the outcome of the receptor engagement in the Treg cell warrants further investigation.

Tumour‐associated Treg cells are known to express numerous co‐stimulatory (i.e. ICOS, OX40, GITR) and co‐inhibitory (i.e. Lag‐3, Klrg1, Tim‐3, TIGIT, PD‐1) receptors that modulate their function (Fig 2). Resolving the functional role of such receptors is important, but is complicated by their frequent expression on conventional T and other immune cell‐types. Hence, lineage‐specific deletion experiments are often required to fully understand their role in cancer. Many potential therapeutic strategies targeting these co‐receptors are primarily aimed at promoting effector T cells in cancer but could induce changes that either help or hinder the therapeutic response, highlighting the importance of considering their role on tumour‐associated Treg cells.

##### Co‐stimulatory receptors

Inducible T‐cell co‐stimulator (ICOS) is important in Treg cell homeostasis and function,^99^, ^100^, ^101^ and is highly expressed by activated Treg cells in prostate cancer^77^ and melanoma.^102^ In the context of cancer, ICOS ligand (ICOSL) can be expressed by tumour cells^103^ as well as myeloid infiltrates, primarily antigen‐presenting cells, including transformed follicular lymphoma B cells^104^ and dendritic cells.^105^ These findings, in conjunction with studies on the function of ICOS/ICOSL signalling in non‐tumour settings, suggest that this pathway is instructive in supporting local Treg cell expansion and function.

The tumour necrosis factor receptor superfamily (TNFRSF) members, including GITR, OX40, CD27 and 4‐1BB are important co‐stimulatory molecules on T cells. In addition to influencing Treg cell development in the thymus, TNFRSF members are critical for the maintenance of Treg cells in peripheral tissues, in part via nuclear factor‐*κ*B/RelA‐mediated signalling.^106^

GITR is highly expressed by Treg cells, and plays an important role in Treg cell expansion.^107^ However, GITR signalling has been proposed to impair Treg cell suppressive capacity, although interrogation of this receptor is complex because of concurrent effects on conventional CD4^+^ and CD8^+^ T cells.^108^ As for ICOSL, GITR ligand (GITRL) is mainly expressed by antigen‐presenting cells, but also endothelial and neuronal cells.^109^, ^110^ In the context of cancer, *in vitro* studies show that tumour‐derived TGF‐*β* can induce GITRL on dendritic cells, which subsequently support expansion of Treg cells.^111^ Interestingly, an association was observed between single nucleotide polymorphisms in GITR (and OX40) and poor survival in ovarian cancer.^112^

OX40 is expressed constitutively by a subset of Treg cells, but also on activated non‐Treg cells.^113^ In cancer, Treg cells can comprise a significant proportion of tumour‐resident OX40^+^ cell types.^114^, ^115^, ^116^ Although OX40‐agonistic reagents are used to stimulate anti‐tumour T‐cell responses, the effect on Treg cells in cancer is not well understood. In wild‐type mice, OX40 is important for Treg cell development in the thymus, as well as expansion in the periphery, although its effect on Treg cell function is less clear. Administration of agonistic reagents demonstrates that OX40‐signalling reduces Treg cell suppressive function *in vitro* and *in vivo*;^117^ however, other studies suggest that Treg cells are not impaired.^118^ One potential explanation is that the context of OX40 ligand (OX40L) expression can have different effects on Treg cells.^113^ In the context of cancer, OX40L is expressed by glioblastoma, especially under hypoxic conditions. *In vitro* experiments indicated that OX40L expression promoted OX40‐driven activation of Treg cells.^119^ In another study of patients with hepatocellular carcinoma, liver‐resident monocytes and macrophages expressed OX40L (in response to concurrent hepatitis C virus infection), which in turn promoted liver Treg cell expansion.^120^ Moreover, two studies identified an association between single nucleotide polymorphisms in OX40L and increased occurrence of breast cancer.^121^, ^122^

4‐1BB is expressed by both lymphoid and myeloid cell types and among T cells, it is expressed by Treg cells and activated CD4^+^ and CD8^+^ T cells.^123^ 4‐1BB activation provides a potent stimulus for anti‐tumour natural killer, CD4^+^ and CD8^+^ T cells and 4‐1BB agonistic antibodies can provoke rejection of tumours in multiple mouse models but non‐specific agonism results in generalized T‐cell activation, cytokine release and systemic inflammation.^123^ Despite initial signs of efficacy, clinical development of agonistic clinical 4‐1BB agonists urelumab and utomilumab has been hampered by inflammatory liver toxicity at moderate systemic doses.^124^ Similar to OX40, 4‐1BB is expressed both on Treg cells and activated CD4^+^ and CD8^+^ Tconv cells. Consequently, it has been difficult to distinguish the effect of therapy on either subset alone and there is contradictory evidence for the effect of 4‐1BB ligation on Treg cells, with some studies demonstrating an inhibitory effect on their immunoregulatory function^125^, ^126^ and others suggesting a stimulatory effect on proliferation.^127^, ^128^ Hence, there is a need to dissect the effect of 4‐1BB agonists on Treg and Tconv cells both in the context of anti‐tumour immunity and the on‐target toxicity that they provoke.

CD27 is expressed mainly by naive and subsets of memory CD4^+^ and CD8^+^ T cells, as well as Treg cells.^113^ CD70 can be expressed by antigen‐presenting cells, but also by tumour‐infiltrating Treg and effector T cells.^129^ Engagement of CD27 with CD70 within tumours is important for expansion of Treg cells within tumours.^129^ Intriguingly, it was proposed that this activity is indirect, and acts through the ability of CD27‐CD70 signalling to drive IL‐2 production by Tconv cells, although evidence supporting this hypothesis was derived from *in vitro* experiments.

In summary, it is clear that co‐stimulatory receptor signalling is critical not only for anti‐tumour immunity, but also influences local Treg cell biology. It is likely that contact‐mediated regulation of Treg cells varies greatly depending on tissue‐ and tumour‐type, and the presence of a shared niche containing cell‐types that express co‐stimulatory ligands.

##### Co‐inhibitory receptors

The co‐inhibitory receptor T‐cell immunoglobulin and ITIM domain (TIGIT) marks a population of Treg cells with enhanced suppressive capacity in tumours.^130^, ^131^ Interestingly, TIGIT^+^ Treg cells preferentially suppress Th1 and Th17 (but not Th2) cells, the former being important in anti‐tumour immunity. Moreover, TIGIT competes for its ligands CD112 and CD155 with the co‐stimulatory receptor CD226 which, unlike conventional T cells, is associated with functionally suppressed Treg cells.^132^ This study suggests that the CD226/TIGIT ratio correlates with Treg cell stability, and clinical outcome in individuals with melanoma. Moreover, both CD112 and CD155 over‐expression is reported in cancer,^133^ although further work is required to delineate the interaction dynamics with the multiple TIGIT‐expressing cell‐types.

Although the role of the inhibitory receptor programmed‐death 1 (PD‐1) on conventional T cells is well established, its function in Treg cells is less clear. Work by the Sharpe laboratory demonstrated that PD‐1 and its ligand PD‐L1 are important for pTreg cell development and function.^134^ Subsequently, PD‐1 was demonstrated to contribute to tTreg cell stability in Foxp3^low^ conditions.^135^ Moreover, PD‐L1 expression on antigen‐presenting cells can expand Treg cells in patients after allogeneic bone marrow transplants.^136^ However, in the context of cancer, PD‐1 expression by Treg cells has been associated with their dysfunction.^137^

The inhibitory receptor lymphocyte activation gene 3 (Lag‐3) is expressed on Treg cells, in addition to other lymphocytes, and binds to two known ligands (MHC‐II and LSECtin). Lag‐3 also promotes suppressive function of Treg cells in homeostasis and cancer.^138^, ^139^ In addition to Treg cell modulation, Lag‐3/MHC‐II interactions are proposed to suppress the maturation of MHC‐II^+^ dendritic cells.^140^ Moreover, LSECtin expression has been reported on tumour cells, suggesting potential Lag‐3‐dependent mechanisms of Treg cell regulation.^141^ T‐cell immunoglobin and mucin domain 3 (Tim‐3) is a co‐inhibitory receptor expressed by different myeloid and lymphoid cells, including Treg cells, and binds to several identified ligands (i.e. galectin‐9, HMGB1, caecam‐1, phosphatidyl serine, reviewed in ref. 142). Tim‐3^+^ Treg cells are detected in many murine and human tumour samples, and have increased suppressive capacity. Conversely, Tim‐3 ligands are widely expressed on both immune and non‐immune cell types in cancer and homeostasis. Another co‐inhibitory receptor, killer cell lectin‐like receptor G1 (Klrg1) is expressed by many immune cells including Treg cells, and binds to its ligands (E‐ and N‐cadherin). Although Klrg1 is used to identify Treg cell subsets, little is known about its functional role. In the gut, Klrg1‐signalling on Treg cells impairs their suppressive function.^143^ Interestingly, loss of E‐cadherin has been shown in cancer progression, suggesting a potential mechanism by which tumour cells promote Treg cell‐mediated immunosuppression.^144^

In summary, cell‐to‐cell interactions via co‐stimulatory or co‐inhibitory receptors are important for local regulation of Treg cell function in cancer. Importantly, the function of these receptors also frequently differs in Treg cells compared with Tconv cells. Moreover, their expression often overlaps with other immune cell‐types, highlighting the need for careful dissection of functional effects on Treg cells.

##### Cytokines

###### Interleukin‐2

Treg cells are mainly dependent upon IL‐2 signalling for both their thymic and peripheral differentiation and for their survival, although IL‐15 and IL‐7 can partially substitute for IL‐2 in maintaining Treg cell survival in the genetic absence of IL‐2^145^or following Treg‐specific disruption of CD25 expression,^146^ respectively. This leads to the question of whether IL‐2 production within tumours is required for maintenance of intratumoural Treg cell populations and what the dominant cellular source of IL‐2 is within tumours (reviewed in ref. 147). There is contradictory evidence regarding the role of IL‐2 in intratumoural Treg homeostasis. Consistent with a role for IL‐2 in tumour immunosuppression, IL‐2 neutralization has been shown to retard the growth of implanted renal cell carcinoma tumours.^148^ Administration of IL‐2 to mice bearing syngeneic B16 melanoma tumours results in an increase in the frequency of Foxp3^+^ cells within the intratumoural CD4^+^ T‐cell pool.^149^ However, the frequency of Treg cells in tumours of mice bearing methylcholanthrene‐induced fibrosarcomas was not affected by administration of IL‐2/anti‐IL‐2 complexes though this does not exclude the possibility that endogenous intratumoural IL‐2 levels are already at functionally saturating levels in such tumours or that other limits to the size of the Treg pool prevent further Treg cell expansion.^90^ In humans, whereas high‐dose IL‐2 therapy is an established treatment for metastatic melanoma and can drive striking clinical responses in a small subset of patients, IL‐2 therapy can also drive expansion of ICOS^+^ Treg cells, the extent of which correlates with worse clinical outcomes following therapy.^150^

In general, CD4^+^ and CD8^+^ Tconv cells are the major cellular source of IL‐2 *in vivo* although the cytokine is also expressed by B cells and dendritic cells.^151^ It is important to note that not all Tconv cells produce IL‐2. Cells in the early stages of differentiation, such as naive and memory CD8^+^ T cells, produce IL‐2 upon stimulation whereas cells that have undergone full effector differentiation do not.^147^ Indeed, loss of the ability of Tconv populations to produce IL‐2 upon full effector differentiation is associated with a catastrophic decline in Treg cell numbers during infection of wild‐type mice with *Toxoplasma gondii* and of perforin‐deficient mice with lymphocytic choriomeningitis virus.^152^, ^153^ Given this exclusively supportive role of Tconv cells in the early stages of differentiation to Treg cell survival, it is important to test whether such cells are present and functionally relevant within tumours. Indeed, a proportion of CD8^+^ T cells within tumours have an early memory phenotype^154^, ^155^ and it will be interesting to determine whether these cells produce IL‐2 and whether similar early memory cells contribute to intratumoural Treg cell maintenance using mouse models. Given the ability of IL‐15 and IL‐7 to act as surrogates for the absence of IL‐2 signalling, it would also be interesting to determine whether these cytokines play a role in Treg cell maintenance in tumours. Finally, Treg cells differ in their requirement for IL‐2 signalling, with CD25^lo^ Treg cells less dependent and intrinsically short‐lived compared with CD25^hi^ cells whose longevity requires CD25 signalling. Hence, the role of IL‐2 will need to be considered in the context of heterogeneity of Treg cell populations and differential requirements for IL‐2.

###### Interleukin‐33

Accumulating evidence indicates that IL‐33 is an important homeostatic factor for Treg cells in multiple tissue sites, in line with their expression of the IL‐33 receptor ST2. Direct action of IL‐33 on Treg cells has been shown to enhance their expansion in the colonic lamina propria, or in the visceral adipose compartment for example.^156^, ^157^ Interleukin‐33 is mostly produced by non‐haematopoietic cells, including fibroblasts and epithelial cells, but also by some activated myeloid cells. Although the precise mechanism remains unclear, it is believed that IL‐33 is released from cells upon certain types of cell death. IL‐33 further functions as an alarmin through the binding to its receptor complex, composed of ST2 and IL1RAcP, expressed on a variety of immune cells, so including Treg cells. In cancer, the role of IL‐33 remains controversial, with both pro‐tumorigenic and anti‐tumorigenic effects reported across different cancer types and cellular sources. Whether the IL‐33/ST2 axis on Treg cells plays a role in tumour progression has yet to be established. Interestingly, administration of IL‐33 to tumour‐bearing mice was shown to expand tumour‐infiltrating Treg cells,^98^, ^158^ in line with the aforementioned IL‐33‐driven expansion of Treg cells in healthy tissues. Recent generation of *Foxp3‐Cre* × *Il1rl1* ^*fl/fl*^ mice^159^ should shed light on the relevance of this pathway *in vivo*.

##### Metabolic fitness

Co‐stimulatory ligands, cytokines and chemokines are well‐established contributors to tTreg cell preferential expansion/maintenance in the tumour microenvironment, but recent evidence points also towards a peculiar cell‐intrinsic metabolism as a means by which tTreg cells survive, expand and exert their function within tumours.^160^ Importantly, tumour‐infiltrating tTreg cells display high expression of the glucose transporter Glut1 compared with splenic Treg cells, and are capable of increased glucose uptake in mouse tumour models.^161^, ^162^ Such an improved glucose usage may in turn fuel fatty acid biosynthesis, in line with observations of a tTreg high neutral lipid content.^162^ Given the important role of both the glycolytic and lipid pathways in tTreg proliferation, suppressive function and trafficking,^160^, ^163^, ^164^ it is tempting to speculate that the adaptation of Treg cell metabolism to ensure exquisite function at the expense of other T cells within the tumour bed is a feature that may be promoted under the influence of tumour and/or stromally derived factors. Identification of such specific cues and pathways may reveal promising therapeutic targets in the near future.

## Future direction and conclusion

Our understanding of Treg cells is rapidly evolving, addressing both long‐standing questions in Treg cell ontogeny and antigen‐specificity while simultaneously exploring new frontiers in the realm of tissue‐residency and interactions with non‐haematopoietic cell types. These new findings may have significant impact in our understanding of tumour immunology, given the clear evidence of Treg cell enrichment, association with poor prognosis, and immune‐regulatory functions in cancer. Importantly, it is clear that Treg cell identity and function are influenced by their niche. In conclusion, our understanding of how Treg cells are themselves regulated will be essential to design novel immunotherapies and leverage existing cancer treatments with greater effect.

## Disclosures

None.
